# Design and current status of CONTINT: continuous versus interrupted abdominal wall closure after emergency midline laparotomy - a randomized controlled multicenter trial [NCT00544583]

**DOI:** 10.1186/1745-6215-13-72

**Published:** 2012-05-30

**Authors:** Nuh N Rahbari, Phillip Knebel, Meinhard Kieser, Thomas Bruckner, Detlef K Bartsch, Helmut Friess, Andre L Mihaljevic, Josef Stern, Markus K Diener, Sabine Voss, Inga Rossion, Markus W Büchler, Christoph M Seiler

**Affiliations:** 1 Department of General, Visceral and Transplant Surgery, University of Heidelberg, Heidelberg, Germany; 2 Institute of Medical Biometry and Informatics (IMBI), University of Heidelberg, Heidelberg, Germany; 3 Department of Surgery, Philipps-University Marburg, Marburg, Germany; 4 Department of Surgery, Technische Universität München and CHIR-Net Munich, Munich, Germany; 5 Department of Surgery, St.-Josefs-Hospital Dortmund, Dortmund, Germany; 6 Study Center of the German Surgical Society (SDGC), Heidelberg, Germany

## Abstract

**Background:**

The optimal strategy for abdominal wall closure has been an issue of ongoing debate. Available studies do not specifically enroll patients who undergo emergency laparotomy and thus do not consider the distinct biological characteristics of these patients. The present randomized controlled trial evaluates the efficacy and safety of two commonly applied abdominal wall closure strategies in patients undergoing primary emergency midline laparotomy.

**Methods/design:**

The CONTINT trial is a multicenter, open label, randomized controlled trial with a two-group parallel design. Patients undergoing a primary emergency midline laparotomy are enrolled in the trial. The two most commonly applied strategies of abdominal wall closure after midline laparotomy are compared: the continuous, all-layer suture technique using slowly absorbable monofilament material (two Monoplus® loops) and the interrupted suture technique using rapidly absorbable braided material (Vicryl® sutures). The primary endpoint within the CONTINT trial is an incisional hernia within 12 months or a burst abdomen within 30 days after surgery. As reliable data on this primary endpoint is not available for patients undergoing emergency surgery, an adaptive interim analysis will be conducted after the inclusion of 80 patients, allowing early termination of the trial if necessary or modification of design characteristics such as recalculation of sample size.

**Discussion:**

This is a randomized controlled multicenter trial with a two-group parallel design to assess the efficacy and safety of two commonly applied abdominal wall closure strategies in patients undergoing primary emergency midline laparotomy.

**Trial registration:**

NCT00544583

## Background

In Germany, more than 700,000 open abdominal operations (laparotomies) are performed each year [1]. A major surgical complication after laparotomy is abdominal fascia dehiscence, which may appear either as an early (burst abdomen with evisceration) or a late (incisional hernia) complication. These patients usually undergo surgery for secondary fascial closure, which is associated with markedly increased morbidity [2], including high recurrence rates (up to 45%) [3]. The applied surgical strategy for abdominal wall closure (that is, the combination of suture technique and material) is of high relevance for the prevention of fascia dehiscence and, moreover, constitutes the main factor directly controllable by the surgeon. However, a recent cross-sectional study among surgeons at institutions participating in a large multicenter trial revealed a lack of consensus regarding abdominal wall closure strategies [4]. In particular, there is uncertainty regarding optimal abdominal wall closure in patients undergoing an emergency laparotomy. Several randomized controlled trials (RCTs) as well as meta-analyses have addressed the issue of optimal fascia closure in elective laparotomies [2,5-12]. Yet, there have been no RCTs dealing exclusively with the emergency setting. As a result, abdominal fascia closure is performed according to the surgeon’s individual preference rather than according to evidence-based data. The present RCT is designed to compare the most established strategies (continuous slowly absorbable sutures and interrupted rapidly absorbable sutures) for abdominal wall closure after midline incisions for emergency laparotomy to determine the superiority of either strategy regarding the development of incisional hernia.

## Methods and design

**Figure 1:**
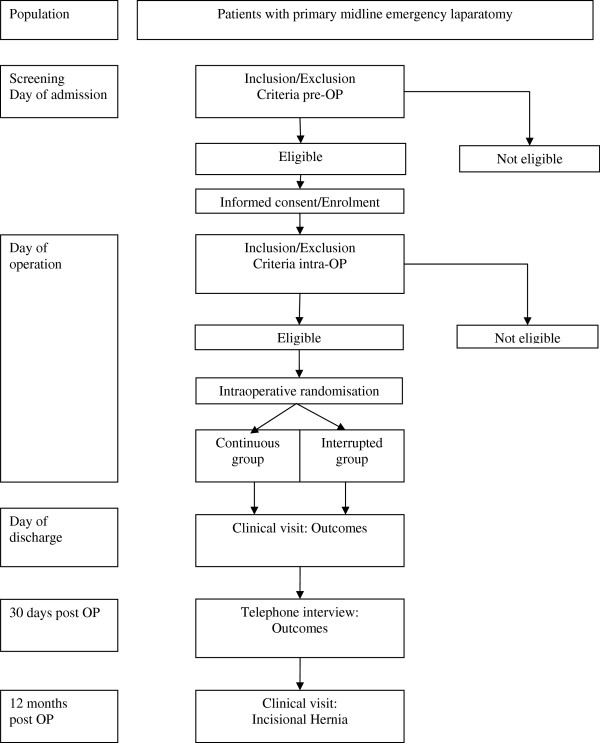
Flow chart of the CONTINT trial.

The CONTINT trial is a randomized controlled two-group parallel superiority trial. Figure 1 shows the study flow chart. Patients requiring an emergency primary midline laparotomy are screened for inclusion into the trial and randomized intraoperatively. The patients’ demographic data and medical history, antibiotic prophylaxis and/or therapy, intraoperative findings, cause of peritonitis, surgical management and source control will be reported. Furthermore, the time needed for fascial closure, length of fascia incision, length of remaining suture material (continuous group) or number of sutures needed (interrupted group) and the surgeon performing the abdominal wall closure will be documented.

In total, five visits are scheduled within the CONTINT trial (Table 1). Follow-up visits are carried out on day 30 after surgery (visit 4) and 12 months after surgery (visit 5). Follow-up data for visit 4 are collected by telephone interview only. Patients are asked study-related questions on pulmonary infection, wound infection or hernia. If necessary, the general practitioner carries out a clinical and ultrasound examination to detect a bulging hernial sac and palpable fascia gap. Quality of life is assessed using a validated questionnaire (SF 36® Health Survey), which has previously been used in trials on surgical interventions. On visit 5, a clinical and ultrasound examination is performed to assess the primary endpoint.

**Table 1:** Course of examinations during the CONTINT trial

**Visit**	**1 (Screening)**	**2 (Operation)**	**3 (Day of discharge)**	**4 (30 days post operation)**	**5 (12 ± 1 months post operation)**
Demographics^a^ and baseline clinical data^b^	X	X			
Eligibility criteria	X	X			
Randomization, surgical intervention		X			
Clinical visit/telephone interview^c^			X	X	X
Quality of life^d^				X	X
Safety^e^		X	X	X	X

^a^Gender, date of birth, height (cm), weight (kg); ^b^smoking, diabetes mellitus, renal insufficiency, chronic pulmonary impairment, immunosuppressive treatment, anemia, malignant disease, American Society of Anesthesiologists score, Mannheim Peritonitis Index [13], indication, surgical intervention; ^c^clinical visit/telephone interview outcome assessment, ultrasound of abdominal wall (only visit 5); ^d^assessed by the Short Form (36) Health Survey (visit 4: by telephone); ^e^serious adverse events.

### **Patient recruitment**

The study protocol was approved by the Independent Ethics Committee, University of Heidelberg. All patients will be informed about the purpose of the CONTINT trial, the applied surgical strategies, and possible benefits as well as potential risks. Screened patients who have not been enrolled into the trial (including patients unable to give informed consent due to any reason) will be documented in the screening log, including the reason for non-inclusion. In order to be eligible for the trial, patients must be older than 18 years and have given informed consent when undergoing primary emergency abdominal surgery. Their expected survival time should be more than 12 months. Patients requiring primary midline laparotomy for ischemic or infectious disease may be included. While patients with a previous laparotomy are excluded, patients who had previously undergone laparoscopy (except for colon resections) or minor abdominal operations (for example, conventional appendectomy, cholecystectomy, hysterectomy, section) may be included. Furthermore, a septic abdominal focus (for example, perforated stomach ulcer, perforated diverticulitis) must be present.

In addition to these preoperative criteria, the septic source must be successfully controlled and abdominal lavage must be performed prior to intraoperative randomization. When a re-laparotomy is planned intraoperatively (that is, a second look operation), patients may not be included. Patients are excluded from the trial if they are already participating in another trial with interference of the intervention and outcome of this study.

### **Endpoints**

The primary endpoint of the CONTINT trial is a burst abdomen within 30 days postoperatively or an incisional hernia within 12 months after the surgery.

A burst abdomen is defined as postoperatively missing continuity of the abdominal fascia in combination with a wound dehiscence and/or a consecutive relapse operation occurring up to day 30 after surgery.

The endpoint ‘incisional hernia’ is assessed by a physical examination and an abdominal ultrasound examination at the trial center 12 months after the operation. A hernia is present if a fascia gap and a protruding hernia sac are seen on ultrasound and if the clinical examination is consistent with a hernia. To guarantee independence of the outcome assessment, the clinical and ultrasound examination needs to be performed by a board certified surgeon familiar with the examination of the abdominal wall, who has not been involved in the surgical procedure of the patient. Ultrasound examinations have to be carried out by an investigator who has had at least six months training in this method.When a patient’s hernia is confirmed by a surgical intervention for an incisional hernia within 12 months after the index operation, the primary endpoint is achieved even in absence of an ultrasound examination by the operation report.

A set of surgical and nonsurgical parameters will be documented as secondary endpoints within the CONTINT trial (Table 2).

**Table 2:** Secondary endpoints within the CONTINT trial

**Secondary endpoint**	**Definition**
**Length of skin incision**	Length of wound from the upper to the lower pole of the skin (cm) after abdominal wall closure (skin staples in place)
**Length of fascia incision**	Length of wound from the upper to the lower pole of the fascia (cm) after closure of fascia
**Time needed for fascial closure**	From the first stitch to the last knot (min)
**Frequency of re-operation due to burst abdomen**	Surgical intervention indicated due to the occurrence of burst abdomen after intervention
**Frequency of re-operation**	Any laparotomy at any time during the follow-up period
**Frequency of abdominal re-interventions**	Any puncture of the abdominal cavity (for example, computed tomography- or ultrasound-guided drainage) at any time during the follow-up period
**Postoperative pulmonary infection**	Infection of the lung with either evidence of increased infection parameters (C-reactive protein > 2 mg/dl and/or leukocytes > 10,0000 cells/ml) that are not caused by a different pathologic process or evidence of pulmonary infiltration in the chest X-ray, requiring antibiotic therapy. Decrease of lung function testing results (forced expiratory volume in 1 s and vital capacity) by 20% or more in comparison with the baseline lung function testing.
**Duration of artificial respiration**	Time on the respirator postoperatively (days)
**Duration of postoperative hemodialysis**	Time on hemodialysis (days)
**Frequency of wound infection**	Surgical site infections within 30 days divided into superficial and deep incisional infections according to the Centers for Disease Control and Prevention definition [14]
**Duration of vacuum therapy**	From first placement of the sponge to removal of the sponge (days)
**Duration of wound healing in patients with secondary wound healing**	From opening of the wound to complete skin closure (days)
**Time to first bowel movement**	From the day of surgery until day of patient’s first bowel movement
**Quality of life**	SF 36 filled in by the patient 30 days and 12 months after the index operation
**Duration of abdominal drainage via intraoperatively placed drains**	From the day of surgery until the day of removal of the last intraoperatively placed drain (days)
**Duration of closed abdominal lavage**	From the day of surgery until the last day of closed abdominal lavage via the intraoperatively placed drains (days)
**Postoperative duration of hospital stay**	From the day of the operation until the day of discharge (days)
**Postoperative duration of intensive care unit stay**	Time from admission to the intensive care unit until transfer to the regular ward (days)
**Mortality**	Death due to any cause at any time during the follow-up period

### **Standardized treatment**

Patients’ intraoperative and perioperative treatment is standardized except for the closure of the abdominal wall, which is carried out according to the allocated study intervention. Antibiotic prophylaxis and therapy is carried out according to local standards (for example, see [15]).

Electric cautery is used to cut skin, the subcutaneous tissue and the abdominal fascia, carefully avoiding damage to the umbilicus [16]. Opening of the abdomen is performed with scissors and incision of the peritoneum is completed with electric cautery. The performed surgical intervention depends on the underlying disease and is performed in line with local standards. In case of a septic focus (for example, a perforated diverticulitis or perforated gastric or duodenal ulcer), source control and abdominal lavage are essential parts of the procedures. Abdominal drains are placed at the end of surgery. Furthermore, drains may be placed for postoperative continuous lavage. Creation of a stoma is not an exclusion criterion for this trial.

### **Trial interventions**

Before closure of the abdominal wall, the length of the fascial incision is measured (in centimeters)*.* Then, four Mikulicz clamps (or equivalent) are placed at the edges of the abdominal fascia. The expertise of the surgeon (board certified versus no certificate) present at the closure is documented.

In the continuous group, the abdomen is closed by a continuous, all-layer suture using two Monoplus® loops, which are made of a slowly absorbable monofilament material. Monoplus® sutures need to be stretched once by the assisting nurse or operation technician before use to avoid breakage of the material. The first stitch has to be anchored cranial and caudal of the incision. After having closed half of the wound, one end of the loop is cut right below the needle. Then one stitch is made back to the opposite edge of the fascia and both ends are tied with at least four counter-rotating knots. At the caudal end of the wound, the same procedure is done with intersection of both loops at the middle of the incision and overlapping of both suture lines for at least 2 cm. Loops are not to be tied together. For every patient, two loops must be used, irrespective of the length of the wound. After completion of the fascial closure, the loop will be cut directly underneath the needle. The length of the remaining suture material has to be measured.

In the interrupted group, patients will receive abdominal wall closure by interrupted sutures using Vicryl® sutures. Anchoring of stitches cranial and caudal of incision is performed in analogy to the continuous suture group. Suturing of the abdominal wall is performed from either end of the incision to the middle of the incision. The number of sutures used has to be recorded. Once all stitches are done, each suture is tied with at least four counter-rotating knots.

For both groups, the maximum distance between the stitches should be 1.5 cm and the distance from the edge of the fascia should be at least 2 cm. Subcutaneous tissue is not sutured and no subcutaneous drainage is used. The skin is closed with clips and the length of the skin incision (in centimeters) is measured*.*

### **Assessment of safety**

Occurrences of events concerning the primary or secondary endpoints are assessed as endpoints only (not as adverse events) and are explicitly asked for.

The following conditions are expected after the initial operation and will therefore not be classified as a complication: pain, nausea, vomiting, urinary tract infection, hyper- or hypotension, imbalances of blood sugar or electrolytes and other laboratory values out of range, if they are not exceeding the duration and extent that can be expected after surgery.

From the day the patient has signed informed consent until the regular end of the trial at 12 months follow-up or until premature withdrawal of the patient, all serious adverse events (SAEs) must be reported within 5 days after the SAE is known. The SAE form contains the following information: identification of the trial participant, attending physician, description of the SAE (event, beginning and duration, severity, outcome, causality to the intervention of the trial, treatment or interventions taken), date and signature of the attending physician.

Analysis of safety-related data will be based on these SAE reports.

### **Randomization and blinding**

Patients will be randomized to one of the two treatment groups just before closure of the abdominal wall using sealed envelopes. Randomization numbers will be allocated to the two groups in balanced permuted blocks using the random number generator of the validated software SAS by the Institute of Medical Biometry and Informatics (IMBI), Heidelberg, Germany. To avoid any potential of predicting group allocation for patients, the block length is fixed in a separate document that is withheld from the study site. As performed in previous studies, sealed and opaque envelopes are produced and labeled with the randomization number containing a data sheet that states the group allocation for the patient with the respective randomization number [17-19]. Randomization envelopes will be used in consecutive order. To avoid manipulations, a thick black bar was placed on the opposite side of the sheet, exactly at the position where the randomization information is located. Basic characteristics of the patient and day of randomization are documented on the data sheet. Thus, compliance to the randomization scheme may be controlled retrospectively. When the study is finished, all unopened envelopes will be sent back to the data management center where they are compared to the allocated randomization numbers and checked for completeness.

The assessors of the CONTINT trial are blinded to the patients’ study treatment.

### **Data management and monitoring**

All protocol-required information collected during the trial is entered in the case report form (CRF) by the investigator or a designated representative. Investigators are expected to complete documentation as soon as possible after the information is collected, preferably on the same day that a trial participant is seen for an examination, treatment or any other trial procedure. An explanation must be given for all missing data.

The completed CRF must be reviewed and signed by the investigator named in the trial protocol or by a designated sub-investigator. After keeping a copy at the trial center, the original CRF is sent to the IMBI for data entry. Double data entry is carried out at the IMBI to ensure correct transfer of data from the CRF to the database. Completeness, validity and plausibility of data are examined by validating programs that thereby generate queries, which need to be clarified by the investigator. At the end of the trial, the principle investigator will retain the original CRFs. All data are managed and analyzed at the joint unit of the Study Center of the German Surgical Society and IMBI in accordance with the current standard operating procedures of the IMBI.

Monitoring is carried out in accordance with ICH E6 (Good Clinical Practice) and standard operating procedures by the Coordinating Center for Clinical Trials, Heidelberg. Participating centers are activated with an initiation visit by the monitor, who will hand out the prepared investigator site file. All relevant trial issues are discussed and trial personnel is trained. Important issues controlled by the monitor are availability of written informed consent and compliance to inclusion and exclusion criteria, as well as documentation of primary endpoint and safety data. Regular contact by phone and/or email with all participating centers will enable the monitor to control the study progression, check adherence to the study protocol, and discuss problems related to the study. In addition, close-out visits are planned for each center.

### **Statistical consideration and sample size**

#### ***Hypothesis***

Within confirmation analysis, the null hypothesis tested is that there is no difference in the incidence of burst abdomen at 30 days postoperatively or incisional hernia within 12 months after emergency midline laparotomy between the two abdominal wall closure strategies under investigation.

#### ***Interim analysis***

When the study was planned, there existed no pilot data and there was no evidence with respect to the scientific question of the study [20]. Therefore, the values of the parameters required for sample size calculation (overall rate and treatment effect to be expected) were completely unknown. Within a fixed sample size design, it was hence uncertain whether the desired power would be achieved or not. For that reason, the study is performed with an adaptive interim analysis. This design allows early stopping of the trial or, if continued, modification of design characteristics - such as recalculation of the sample size - under control of the global type I error rate.

The implementation of the design is as follows: an adaptive interim analysis as described by Bauer and Köhne [21] will be performed after 80 evaluable patients have completed the 12 months follow-up. The global one-sided type I error rate of the trial is controlled by 0.025. This is achieved by implementing the following decision rules. The study is stopped after the interim analysis with acceptance of the null hypothesis if the one-sided *P*-value lies above 0.40, and with rejection of the null hypothesis if the one-sided *P*-value falls below 0.0115. Otherwise, the study is continued with a second stage, and the null hypothesis is rejected in the final analysis if the product of the stage-wise one-sided *P*-values falls below 0.0038.

#### ***Data analysis***

For the interim analysis as well as for the final analysis, each patient’s allocation to the different analysis populations (full analysis set (FAS), per protocol (PP) analysis set, safety analysis set) will be defined prior to the analysis. The allocation will be documented in the statistical analysis plan. During the data review, deviations from the protocol will be assessed as ‘minor’ or ‘major’. Major deviations from the protocol will lead to the exclusion of a patient from the PP analysis set.

The null hypothesis is assessed by testing the effect of the wall closure procedure (continuous versus interrupted) in a logistic regression model that takes into account the covariates body mass index (BMI; values as measured on the original scale) and age (values as measured on the original scale). The decision rules according to the adaptive design as specified above will be applied in the interim and the final analysis, respectively.

Confirmatory analysis will be primarily based on the FAS, which is consistent with the intention-to-treat principle by including all patients who were randomized to the two groups. This approach reflects the idea that the study should match as closely as possible to the conditions in clinical practice.

If a patient leaves the study prematurely, missing data with respect to the primary outcome variable will be replaced by the Imputed Case Analysis by incorporating available reasons for missing data (ICA-r) method described by Higgins *et al*. [22].

In addition to the evaluation of the FAS, a PP analysis will be performed for the primary endpoint including all randomized patients without major protocol violations. The secondary variables will be analyzed descriptively by tabulation of the measures of the empirical distributions and by application of appropriate tests whose results are to be interpreted in a strictly descriptive sense.

#### ***Determination of the sample size***

The sample size calculation is based on the two-sided chi-square test for difference for the assessment of the primary endpoint. It can be expected that taking the potential confounders BMI and age into account in the analysis will increase the power as compared with the chi-square test. Due to the lack of any empirical data on the overall rate and the treatment effect to be expected, only a preliminary sample size calculation is performed and a sample size reassessment is conducted in a planned adaptive interim analysis (see above). The power for early rejection of the null hypothesis after the first study stage with a total of 80 evaluable patients amounts to 80% if, for example, the true incidence rates of incisional hernia or burst abdomen are 0.20 and 0.53 or 0.30 and 0.65, respectively, in the two groups.

A sample size of 80 evaluable patients until the interim analysis was therefore considered as both feasible and sufficient to gain enough information required for sample size recalculation in case the trial is continued after the interim analysis. The rate of loss to follow-up observed up to the interim analysis will also be taken into account when calculating the sample size for the second stage.

## Discussion

The optimal strategy of abdominal wall closure after midline laparotomy has remained an issue of ongoing discussion. To date, various RCTs and meta-analyses on abdominal wall closure strategies after midline laparotomy have been published with heterogeneous results. A recent meta-analysis identified several RCTs on techniques and materials of abdominal wall closure after elective laparotomy [20]. While some of the detected studies included patients who underwent emergency laparotomy, there were no trials that specifically enrolled patients who had emergency surgery. The pooled analyses demonstrated a benefit of the continuous suture technique on the development of incisional hernia. Remarkably, statistical heterogeneity of the meta-analyses increased substantially if studies that enrolled patients with emergency laparotomy were included, whereas there was almost no heterogeneity in the sensitivity analyses excluding these studies. These findings suggest a significant difference between the biological properties of and demands to the abdominal wall of patients undergoing elective and emergency laparotomy. Reasons for this difference have not been clarified in detail and probably include factors such as a contaminated operative field, poor general conditions of these (septic) patients and different requirements to the abdominal wall, especially in patients requiring prolonged mechanical ventilation. In particular, the proliferation of bacteria in tissue represents a strong risk factor causing wound infection with delayed healing of the wound and wound dehiscence [23]. The adverse impact of emergency surgery on the incidence of postoperative complications as compared to an elective setting has already been demonstrated in various studies [24-28]. In a large cohort study including more than 4.800 patients who underwent open gastrointestinal surgery, emergency surgery was associated with a significantly increased incidence of postoperative tissue and wound complications [24]. On multivariate analysis, male gender, peritonitis, kind of operation and multiple operations were independent predictors of tissue and wound complications in the subgroup of patients who underwent emergency surgery.

Based on the described differences between patients undergoing emergency and elective laparotomy as well as the current lack of studies, there is an urgent need for prospective studies specifically assessing strategies of abdominal wall closure after emergency midline laparotomy The present RCT compares the two most commonly applied strategies of abdominal wall closure after midline laparotomy, that is, the continuous, all-layer suture technique using slowly absorbable monofilament material (two Monoplus® loops) and the interrupted suture technique using rapidly absorbable braided material (Vicryl® sutures). The incidence of burst abdomen by 30 days or incisional hernia within 12 months after surgery was chosen as the primary endpoint to specifically assess the impact of both closure techniques on abdominal fascia healing in the emergency setting. In theory, the interrupted closure technique may allow discharge of (contaminated) ascites and thus be associated with fewer intra-abdominal complications (for example, fluid collections or abscess) as well as improved abdominal fascia healing. However, a randomized trial is required to validly assess superiority of this technique.

An adaptive interim analysis is performed in the CONTINT trial to cope with the lack of reliable data required for a substantiated sample size calculation.

To achieve a homogenous study population, only patients undergoing primary midline laparotomy for an emergency indication will be included in the CONTINT trial. Furthermore, the confirmatory analysis of the overall treatment effect is performed using a logistic regression model that includes patients’ BMI and age as potential confounding factors. There is a recognized need to standardize the definition of complications after surgical interventions to enable comparisons of results from different studies [29-31]. For this reason, endpoints within the CONTINT trial have been defined *a priori* to minimize assessment bias.

In conclusion, the CONTINT trial represents the first RCT assessing efficacy and safety of two different abdominal wall closure strategies in patients undergoing emergency midline laparotomy. Applying high methodological standards, the results of the trial will help to improve and standardize surgical treatment in this specific population of patients that may not be simply compared to patients undergoing elective surgery.

## Trial implementation and status

### Protocol development and trial organization

The CONTINT trial has been designed initially as a single center RCT and was initiated at the Department of Surgery, University of Heidelberg in November 2007. After 1.5 years of recruitment, a total of 16 patients had been enrolled in the study. Due to the insufficient recruitment of patients at a single center, we decided to expand the number of trial institutions, including distribution via the Trial Network of the German Surgical Society (CHIR-*Net*). Consequently, the trial protocol was amended and an investigators’ meeting was held at Frankfurt, Germany on 31 August 2009. Up to this date, 26 patients had been recruited.

A total of 11 trial centers have approved participation in the CONTINT trial. A total of 73 patients have been randomized at the date of submission of this paper.

## Competing interests

The authors declare that they have no competing interests.

## Authors’ contributions

NNR: obtained funding, study design, drafting of the manuscript. PK: study design, revision of the manuscript. MK: sample size calculation, planned statistical analyses, revision of the manuscript. TB: study design, revision of the manuscript. DKB: study design, revision of the manuscript. HF: study design, revision of the manuscript. ALM: study design, revision of the manuscript. JS: study design, revision of the manuscript. MKD: study design, revision of the manuscript. SV: study design, revision of the manuscript. IR: study design, revision of the manuscript. MWB: study design, revision of the manuscript. CMS: obtained funding, study design, revision of the manuscript. All authors have read and approved the final version of the manuscript.

## Participating centers

The CONTINT study group includes the following trial centers: Klinikum Augsburg (M. Anthuber, R. Stiegeler); St.-Josefs-Hospital Dortmund (J. Stern, E. Stein, A. Rönsch); St. Augustinus Krankenhaus Düren (C. Rudroff, S. Hilswicht); Westküstenklinikum Brunsbüttel u. Heide (F. Kallinowski, T. Eibisch); University Hospital Heidelberg (C. Seiler, N. Rahbari, P. Knebel); St. Bonifatius Hospital Lingen (C. Seidlmayer); University Hospital Marburg (D. Bartsch, A. Franck, R. Ramerth); CHIR-*Net* Munich (Surgical Regional Center Munich of the Trial Network of the German Surgical Society, DGCH) at the Klinikum rechts der Isar, Technische Universtität München, Munich, Germany (H. Friess, C. Schuhmacher, Andre L. Mihaljevic; U. Böcher) and at Klinikum Freising (F. Zeller, C. Ruf). CHIR-*Net* Munich is funded by the BMBF (01GH1001C); Krankenhaus Sinsheim (Th. Simon, Th. Löffler) Bethesda-Krankenhaus Stuttgart (B. Kraft, H. Demirdelen).
